# Nigeria bee honey-enhanced adherence, neovascularisation and epithelisation of full-thickness skin autografts on distal extremities of dogs

**DOI:** 10.1186/s12917-022-03192-w

**Published:** 2022-03-11

**Authors:** Dorcas Oyueley Kodie, Noah Segun Oyetayo, Oluwasanmi Olayinka Aina, Oghenemega David Eyarefe

**Affiliations:** 1 Department of Veterinary Surgery and Radiology, Faculty of Veterinary Medicine, University of Ibadan, Ibadan, Oyo State, Nigeria; 2 Department of Veterinary Anatomy, Faculty of Veterinary Medicine, University of Ibadan, Ibadan, Oyo State, Nigeria

**Keywords:** Autograft, Dog, Honey, Skin, Wound

## Abstract

**Background:**

Full thickness skin grafts (FTSGs), although ideal for resurfacing large defects of the distal extremities in veterinary patients, have a high failure rate due to issues of adherence, infection and inadequate revascularisation because of its thickness and high nutritional demand. This study investigated the effect of Nigeria bee honey on FTSG take at the distal extremities of dogs. The study was conducted on 6 adult male Nigerian indigenous dogs using 3 of the 4 limbs of each dog randomly divided into 3 treatment groups: Nigerian bee honey (HON group), platelet-rich plasma (PRP group) and normal saline (CON group). Full‐thickness skin wounds (3 cm × 1.5 cm) were created on the lateral aspect of the radioulnar or metatarsal areas and dressed till adequate granulation tissues formed. Donor skins harvested from the lateral thorax of each dog were sutured to the recipient bed following application of the assigned treatment, and evaluated grossly and histologically on days 0, 4, 7, 10, 14, 17, and 21.

**Results:**

A higher percentage (4/6 representing 66.7%) of complete graft take was observed in the HON and PRP groups as compared to 3/6 (50%) in the CON group. The HON group had a greater percentage (5/6 representing 83.3%) of adhered grafts as compared to the PRP (4/6 representing 66.7%) and CON (3/6 representing 50%) groups at day 4. There was a significant decrease (*p* = 0.022) in percentage necrosis between the CON and HON/PRP groups on day 10, 14 and 17. The percentage open mesh area for the HON group was significantly lesser at day 4, 7 and 10 when compared with CON (*p* < 0.001) and at day 4 when compared with PRP (*p* = 0.001). At histology, graft neovascularisation score was highest in the HON group on days 4, 14 and 21.

**Conclusion:**

Nigeria bee honey enhanced take of meshed full-thickness skin autografts by promoting adherence to the recipient bed, enhancing fibroblast proliferation and collagen laydown, and accelerating the rate of neovascularisation suggesting promising application as an alternative modality to enhance FTSG take.

**Supplementary Information:**

The online version contains supplementary material available at 10.1186/s12917-022-03192-w.

## Introduction

Managing wounds of the extremities is relatively more challenging than other anatomical areas due to the limited amount of skin in the region [1, 2]. Most of these wounds are unamenable to primary closure, and second-intention healing or skin stretching techniques are only possible when the wound involves less than 30% circumference of the limb [3]. When large wounds of the limb’s distal extremities are allowed to heal by second intention, delayed wound contracture occurs which often leaves a large scar, and in some cases, can lead to functional limb deformity [4]. As a result, free skin graft techniques are preferred for such large wounds of the limb’s distal extremities.

Free skin grafts are classified into two main groups: full-thickness skin grafts (FTSGs) and partial/split-thickness skin grafts (STSGs), depending on the layers of skin harvested from the donor site [5–7]. FTSGs typically consist of the entire epidermis and dermis, while STSGs include the entire epidermis and varying sections of the dermis. FTSGs are preferred where good cosmetic outcome or a durable skin cover is necessary [6–8]. Currently, STSGs are the gold standard in human plastic surgery [9–11]. However, FTSGs have some advantages that are priced in veterinary reconstructive surgery which include less contraction on healing, thicker skin that is more resistant to trauma, and better aesthetic outcome after complete healing with good hair regrowth [6, 7].

A major challenge of FTSGs is the greater chance of graft failure attributed mostly to its thickness [12]. Due to the sequence of skin graft healing, that includes adherence, plasmatic imbibition, inosculation and capillary regrowth, and complete reattachment and revascularisation of the graft, STSGs tend to fail less often than FTSGs and heal faster. Graft healing is complete when there is full reattachment and revascularisation of the graft, which when successful is described as graft ‘take’ [13, 14]. This is dependent on the establishment of arterial connections between the graft and the recipient bed, which should occur by the 7th or 8th postoperative day, or the graft is considered to have failed [15].

Due to the tendency of FTSGs to fail, techniques, as well as the use of natural products, for enhancing FTSG take are constantly being studied. The use of vasoendothelial growth factor (VEGF) [16, 17], fibrin glue, platelet-rich plasma (PRP) [18] and other natural binding agents like keratin and gelatine [19] have been documented. Recently, negative pressure or vacuum bandaging has been suggested for early graft adherence [7, 20].

Honey has been widely studied for its anti-inflammatory, tissue regeneration, angiogenic and epithelisation properties [21–26]. Honey’s application in skin grafting, however, has been limited to fixation of STSGs to the recipient bed in humans to facilitate graft adherence in the absence of sutures or staples [27, 28]. Crude Nigeria bee honey has been reported to possess antibiotic and angiogenic properties comparable to that of commercially available medical grade honey [29, 30]. It has been used in recent times to promote healing of various kinds of wounds in both experimental and clinical settings [31–34] with exceptional results. Due to these angiogenic, antibacterial and tissue adhesive properties, it was hypothesised that honey could enhance take of FTSGs by facilitating early adherence and promoting neovascularisation and epithelisation while preventing infection at the wound site. This study therefore investigated the effect of Nigeria bee honey dressing on the survival of FTSGs using indices including adherence to recipient bed, rate of revascularisation of skin graft, and rate of epithelisation, as well as the cellular mechanism(s) by which Nigerian bee honey will influence take of FTSGs at the distal extremities of dogs.

## Results

### Gross Evaluation of Graft Healing

Out of the 18 wounds grafted at the distal extremities of the dogs, 11 (61.1%) had complete graft take, 5 (27.8%) had partial graft take while graft loss occurred in the remaining 2 (11.1%). The HON and PRP groups had higher percentages of complete graft take with 4 out of 6 (66.7%) each as compared to the CON group which had 3 out 6 (50%) complete graft take. Partial graft take was recorded in 2 out of 6 (33.3%) each for the CON and PRP groups, and 1 out of 6 (16.7%) in the HON group while graft loss was recorded in 1 out of 6 (16.7%) each in the CON and HON groups.

### Graft Adherence

Grafts were non-mobile (adhered to the recipient bed) in 3 (50%) and 4 (66.7%) out of 6 in the CON and PRP groups respectively and 5 out of 6 (83.3%) in the HON group on day 4. All 16 grafts with complete or partial graft take were non-mobile by day 7.

### Presence of seroma

Seroma was observed beneath the graft in 3 out of 6 for the CON group and 2 out of 6 for the PRP and HON groups. Seroma was present at both day 4 and 7 in 1 CON graft with all others present on day 4 only. Wound fluid across all the groups was minimal in quantity, pink or brown in colour and serosanguineous in nature on days 4 and 7 with the secondary layer appearing dry and clean by day 10, indicating cessation.

### Graft Colour

**Fig. 1:**
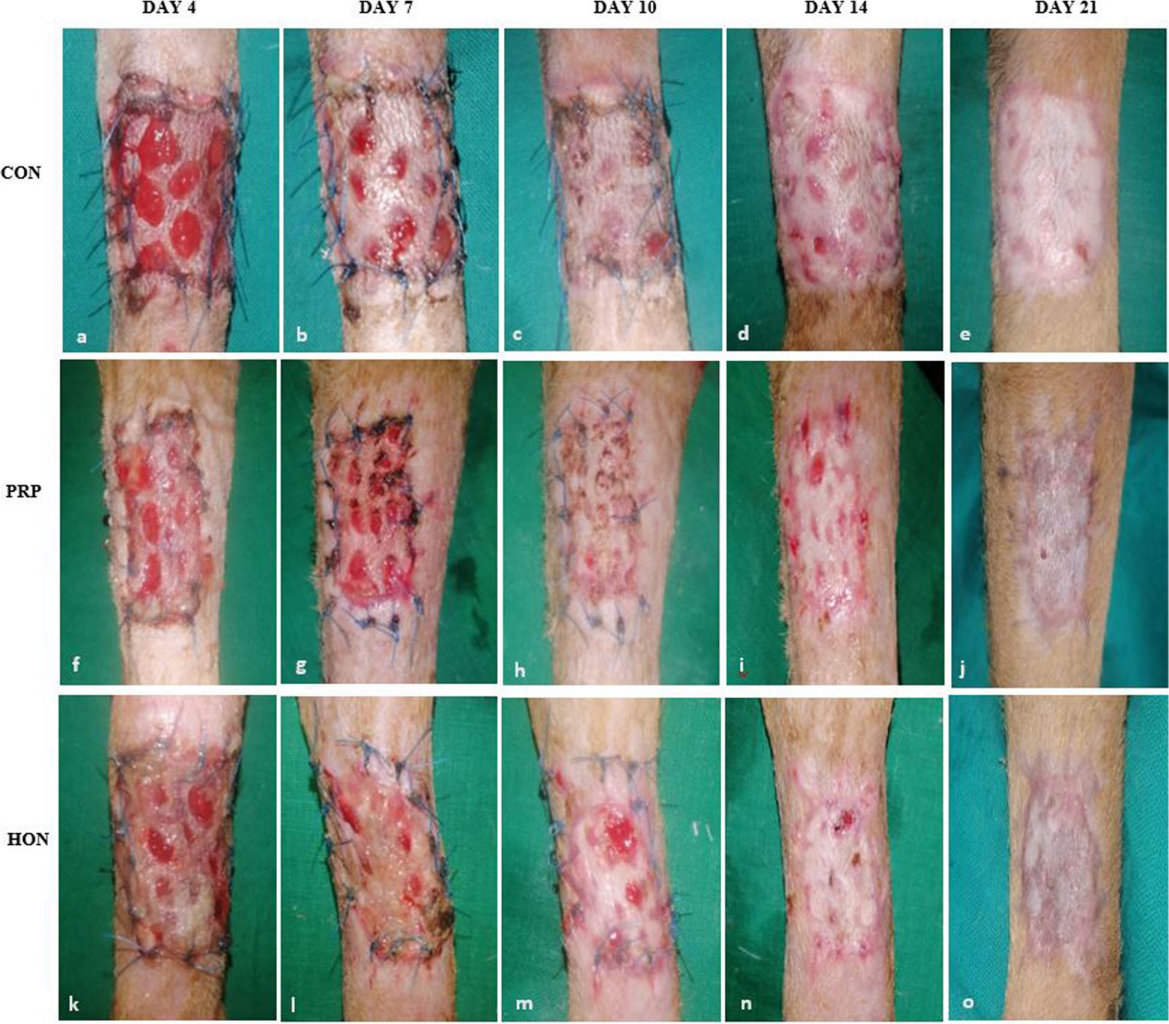
Progressive healing of meshed FTSGs with complete graft take from each treatment group

The mean scores of graft colour at each day of dressing is presented in Table 1. There was gradual change in graft colour from purple or mottled pink to healthy pink in all groups from day 4 to day 21 (Fig. 1), with the change being more rapid in the HON group between day 7 and 10. There was also grossly observable difference in colour (mottled pink to healthy pink) between day 10 and 14 in the CON and PRP groups. The trend observed was HON > PRP > CON. However, by day 17 and 21, all grafts that had complete and partial graft take were similar in appearance, with good epithelial lining cover on areas with initial devitalised tissue.

**Table 1:** Mean scores of graft colour recorded on dressing days

Group	Day 0	Day 4	Day 7	Day 10	Day 14	Day 17	Day 21
CON PRP HON	1 1 1	3.1 3.3 3	3 2.6 2.2	2.7 2.3 1.2	1.5 1.3 1.2	1.2 1.2 1.2	1.2 1.2 1

1 = healthy pink; 2 = mottled pink; 3 = mottled, purple; 4 = dark purple or black; and 5 = slimy white

### Percentage Necrosis

**Fig. 2:**
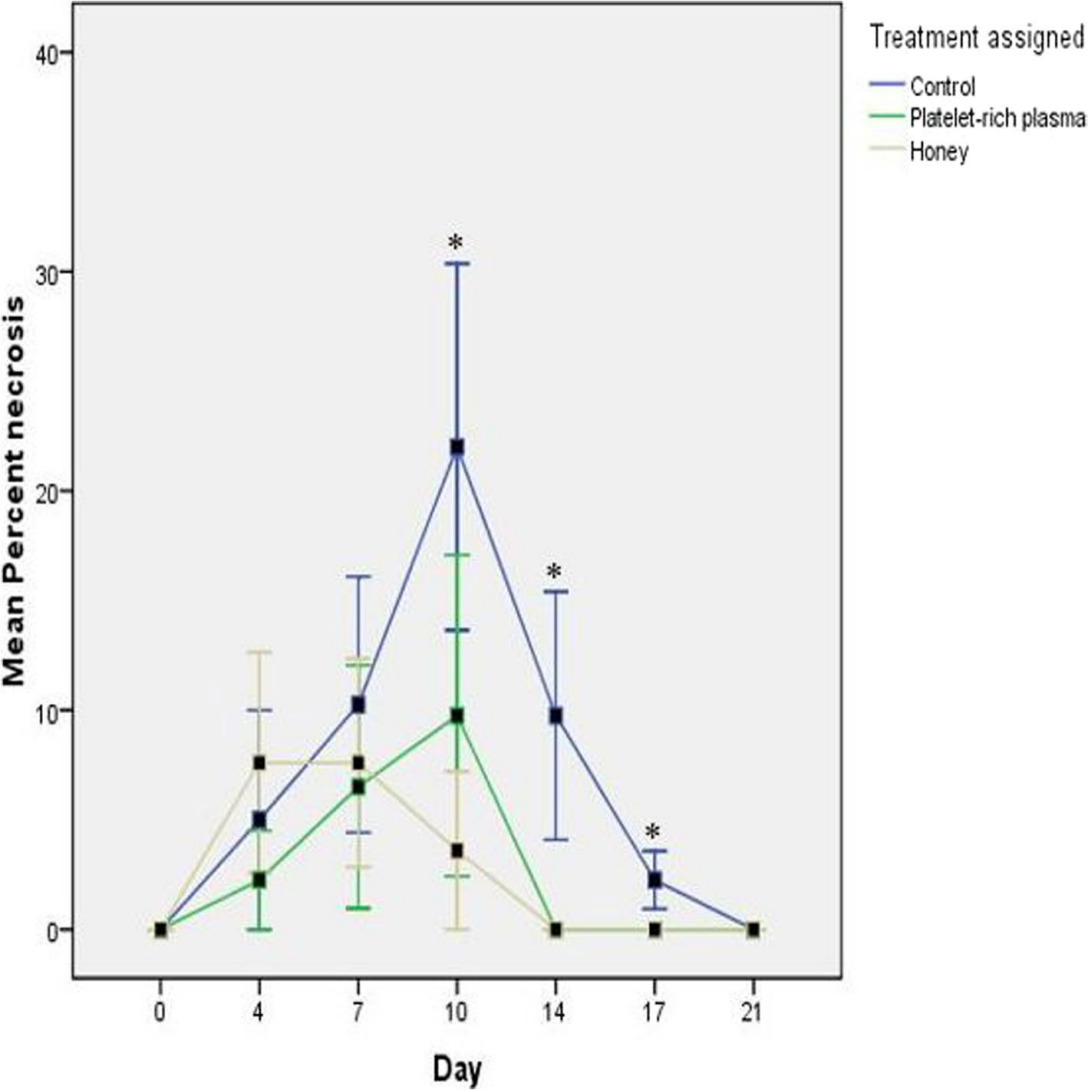
Mean results for percentage necrosis across treatment groups. Line graph with standard error bars (Mean ± SE) showing the mean percentage necrosis of meshed FTSGs treated with normal saline (CON), platelet-rich plasma (PRP) and honey (HON). Asterisks indicate significance (values where *p* < 0.05)

Percentage area of necrosis increased between day 0 and 10 for the CON and PRP groups and between day 0 and 7 in the HON group (Fig. 2). There was a rapid neovascularisation of necrotic areas in the PRP and HON groups by day 14 but the process was gradual in the CON group till day 21. There was a statistically significant decrease in percentage necrosis (*p* = 0.022) between the CON and HON/PRP groups on day 10, 14 and 17 (Fig. 2).

### Percentage Open Mesh Area

**Fig. 3:**
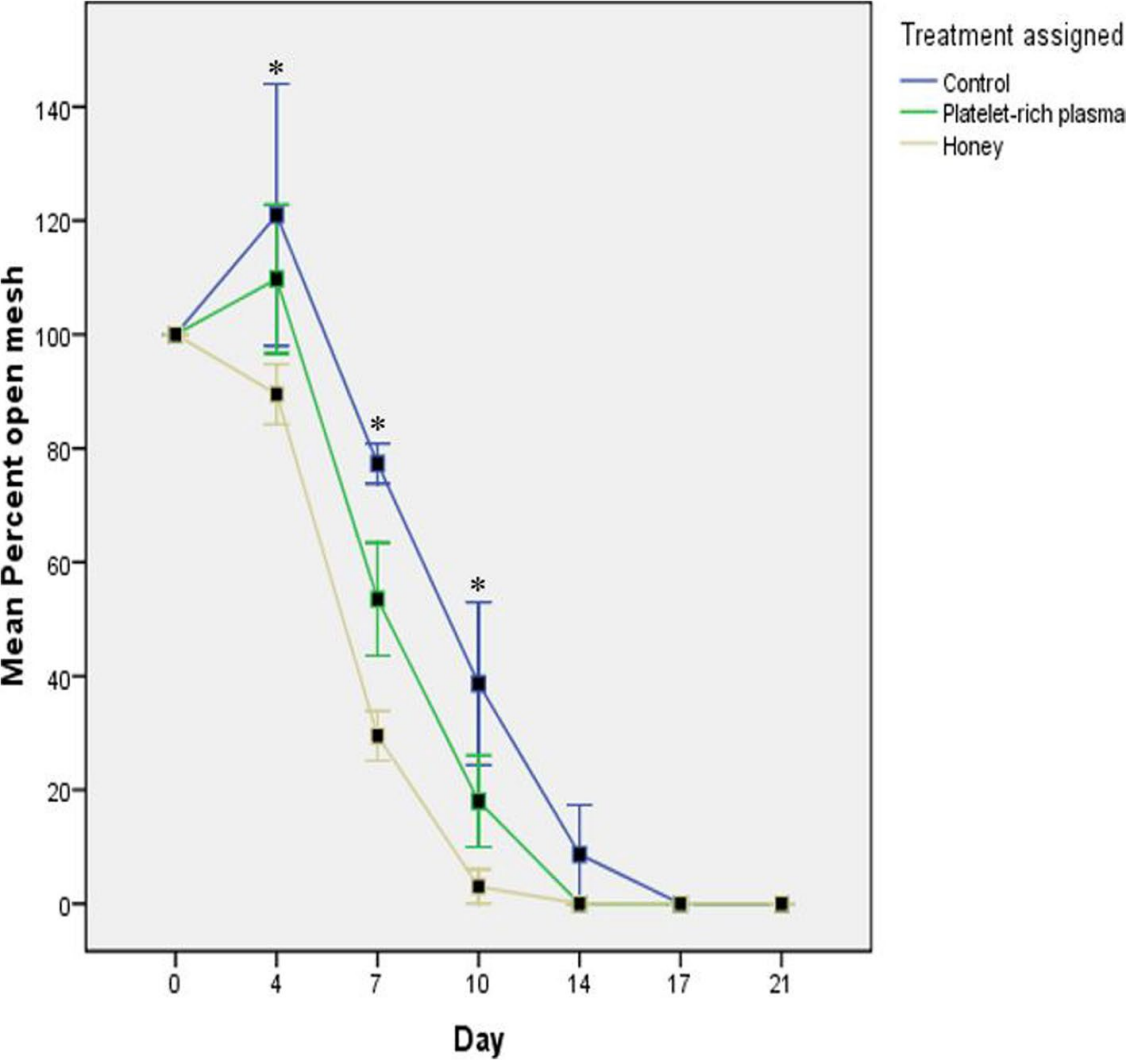
Mean results for percentage open mesh area across treatment groups. Line graph with standard error bars (Mean ± SE) showing the mean percent open mesh area of meshed FTSGs treated with normal saline (CON), platelet-rich plasma (PRP) and honey (HON). Asterisks indicate significance (values where *p* < 0.05)

Percentage area of open mesh increased in the CON and PRP groups on day 4 but decreased in the HON group before steadily reducing across all groups between day 4 and 14 with the trend being HON > PRP > CON. There was a statistically significant difference (*p* < 0.001) in open mesh area between the CON and HON groups on day 4, 7 and 10, and between the CON/PRP and HON groups on day 4 (*p* = 0.001). Re-epithelisation in the HON group was near complete by day 10. Re-epithelisation of grafts was more rapid in the HON and PRP groups between day 4 and 14 in comparison to the CON group which was gradual and complete by day 17 (Fig. 3).

### Final Aesthetic Outcome and Hair Regrowth

**Fig. 4:**
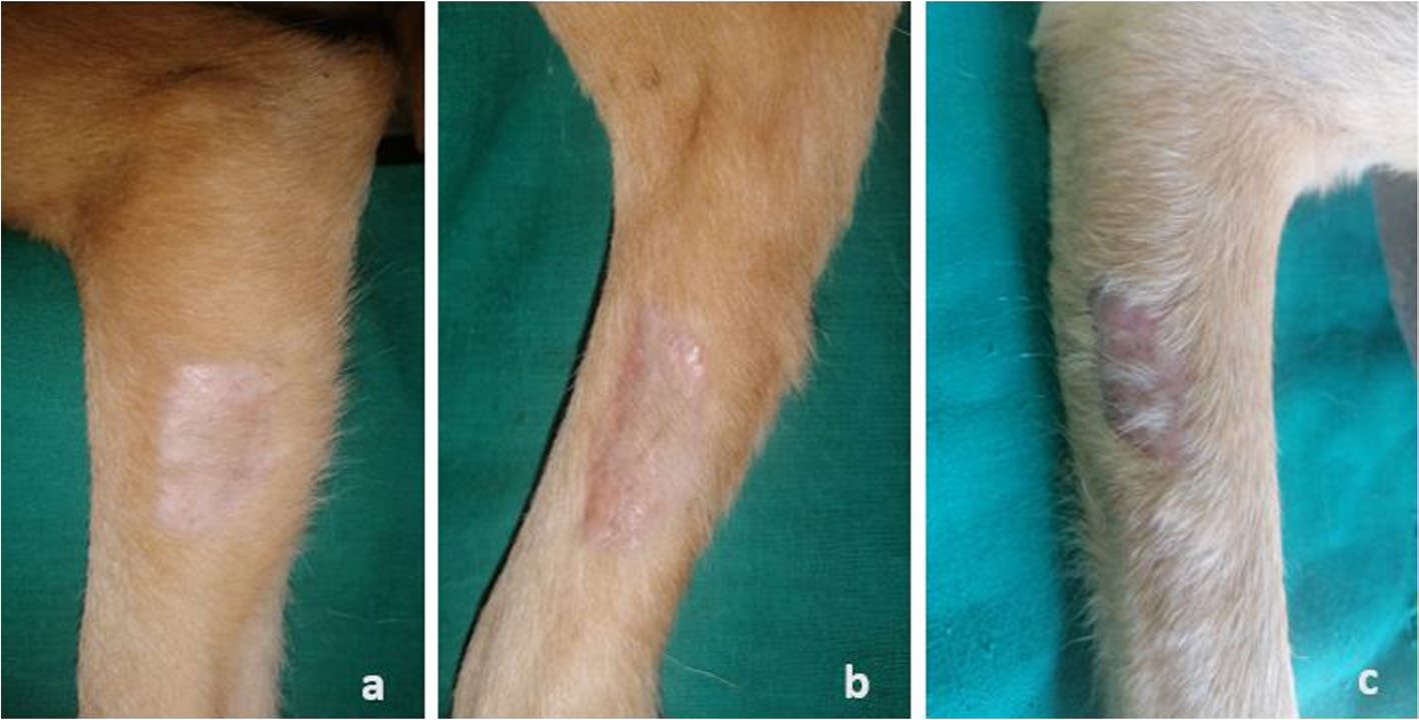
Representative images of meshed FTSGs from each group on day 65 post-grafting. **a **Sparse hair regrowth was observed at the grafted site in the CON grafts. **b **Sparse to good hair regrowth was observed at the grafted site in the PRP grafts. **c **Good hair regrowth was observed at the grafted site in the HON grafts. The absence of hair regrowth at the periphery of the grafted sites was attributed to epithelial covering of the biopsy sites

All grafts with complete or partial take were healed by day 21, assuming the original pigmentation of the donor skin (Fig. 1). Hair regrowth was observed across all the groups (Fig. 4) but was better in the HON and PRP groups in comparison to the CON group.

## Histologic evaluation of graft healing

### Epidermal and Follicular Epithelium Architecture of Grafts

**Fig. 5:**
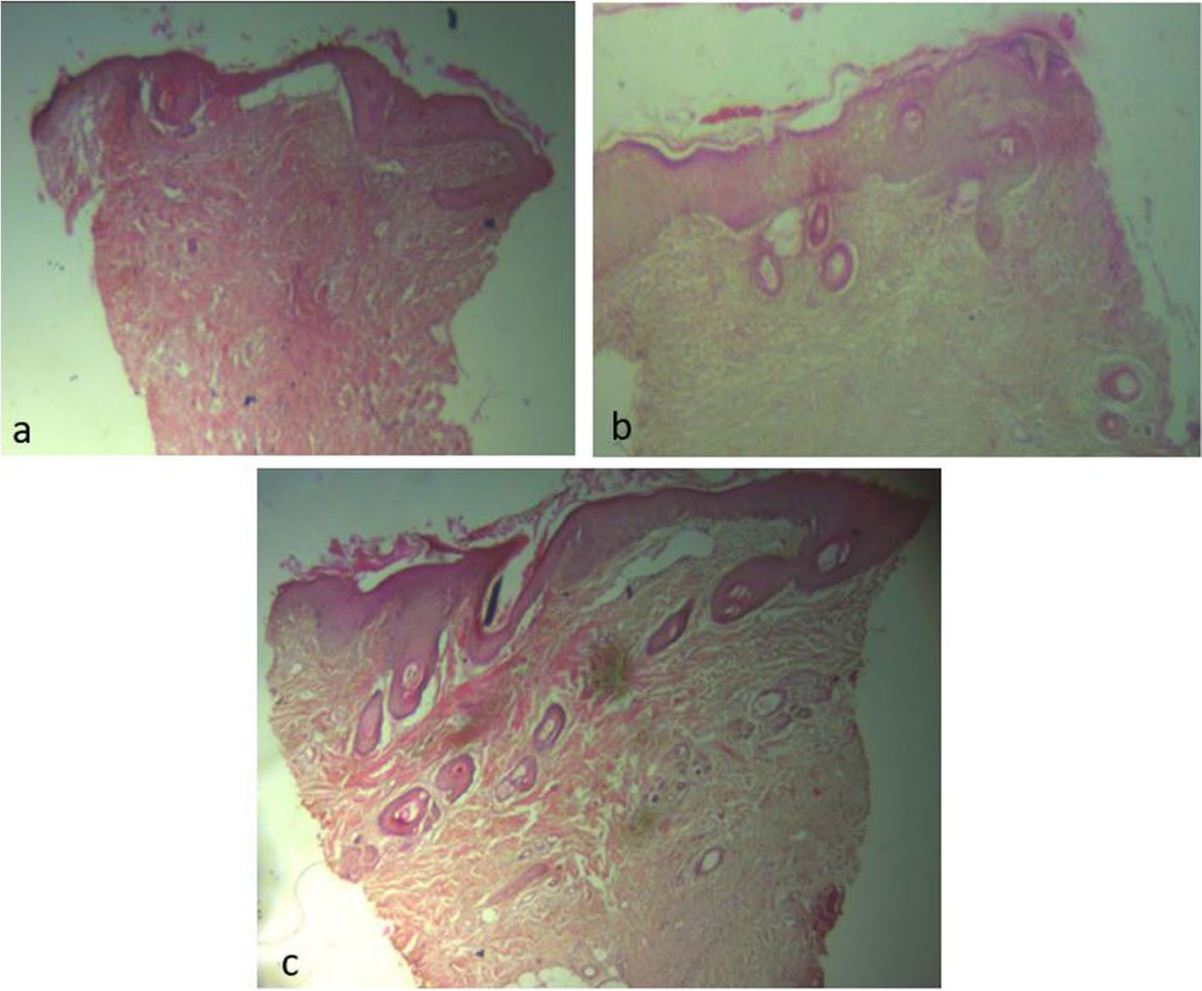
Photomicrograph of biopsy of meshed FTSG taken at day 10. H&E × 40. Full thickness epidermal architecture, including that of follicular epithelium was more well-structured by day 10 in the HON group (**c**) in comparison with the PRP (**b**) and CON groups (**a**)

Epidermal devitalisation (presented histologically as full or partial thickness devitalisation of all keratinocyte layers and the follicular epithelium) was marked in all the 3 groups on day 4, but progressively decreased from day 4 to 14. Full thickness epidermal architecture, including that of follicular epithelium was more well-structured by day 10 in the HON group in comparison to the PRP and CON groups (Fig. 5). However, there was progressive necrosis and thinning of the epidermis in the HON group from day 10 to 21.

### Histologic Assessment of Graft Tissue Inflammation (HAIS) and Repair (HRS)

**Fig. 6:**
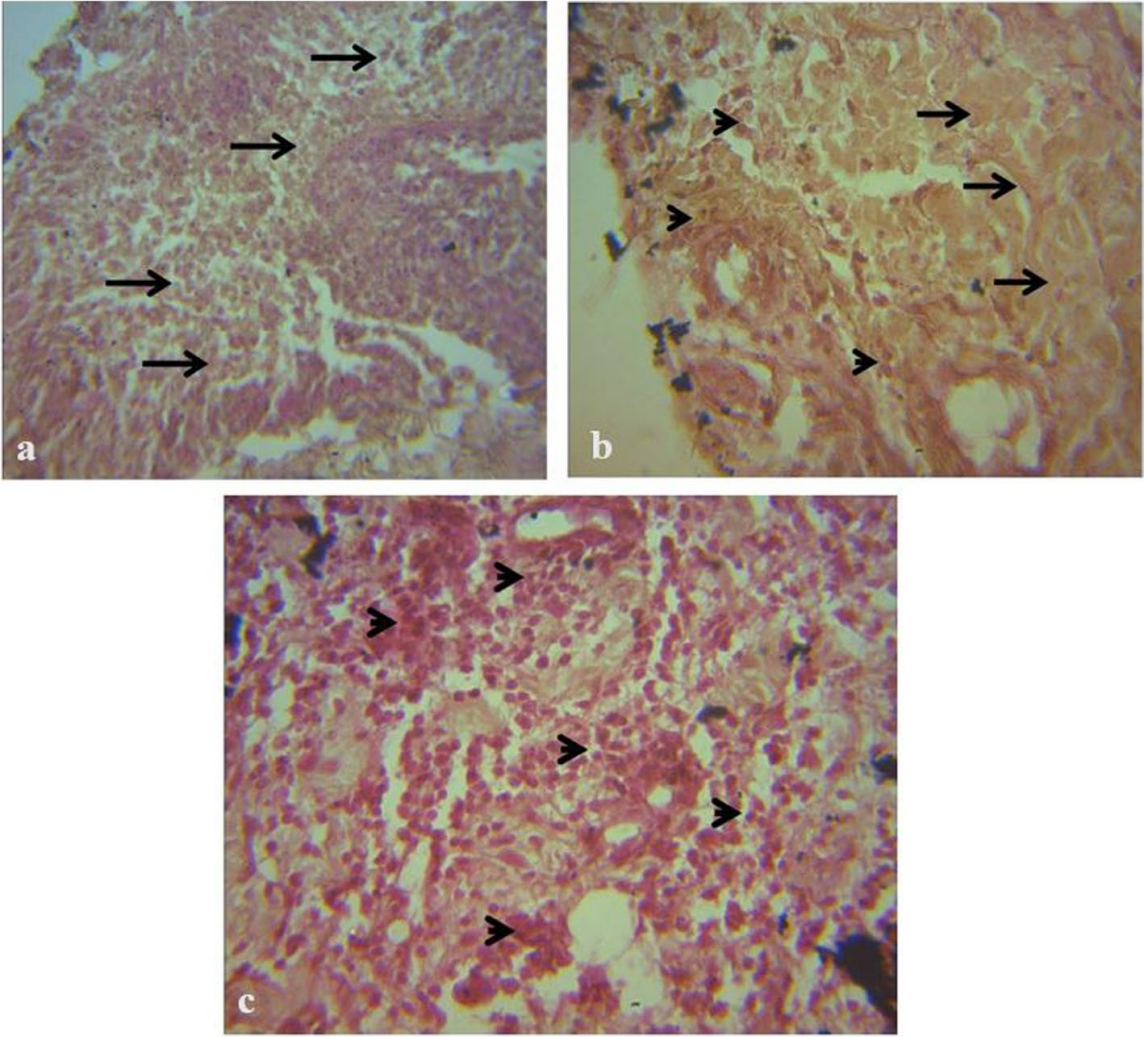
Photomicrograph of biopsy of meshed FTSG taken at day 4. H&E × 400. **a **There is a diffuse moderate necrosis of dermal cells/connective tissue in the CON graft, the field appears haemorrhagic (arrows). **b **There is collagen fibre laydown (arrows), and mild to moderate cellular infiltration made up mostly by macrophages (arrowheads) in the PRP graft. **c **There is a marked cellular infiltration (arrows) in the HON graft

**Fig. 7:**
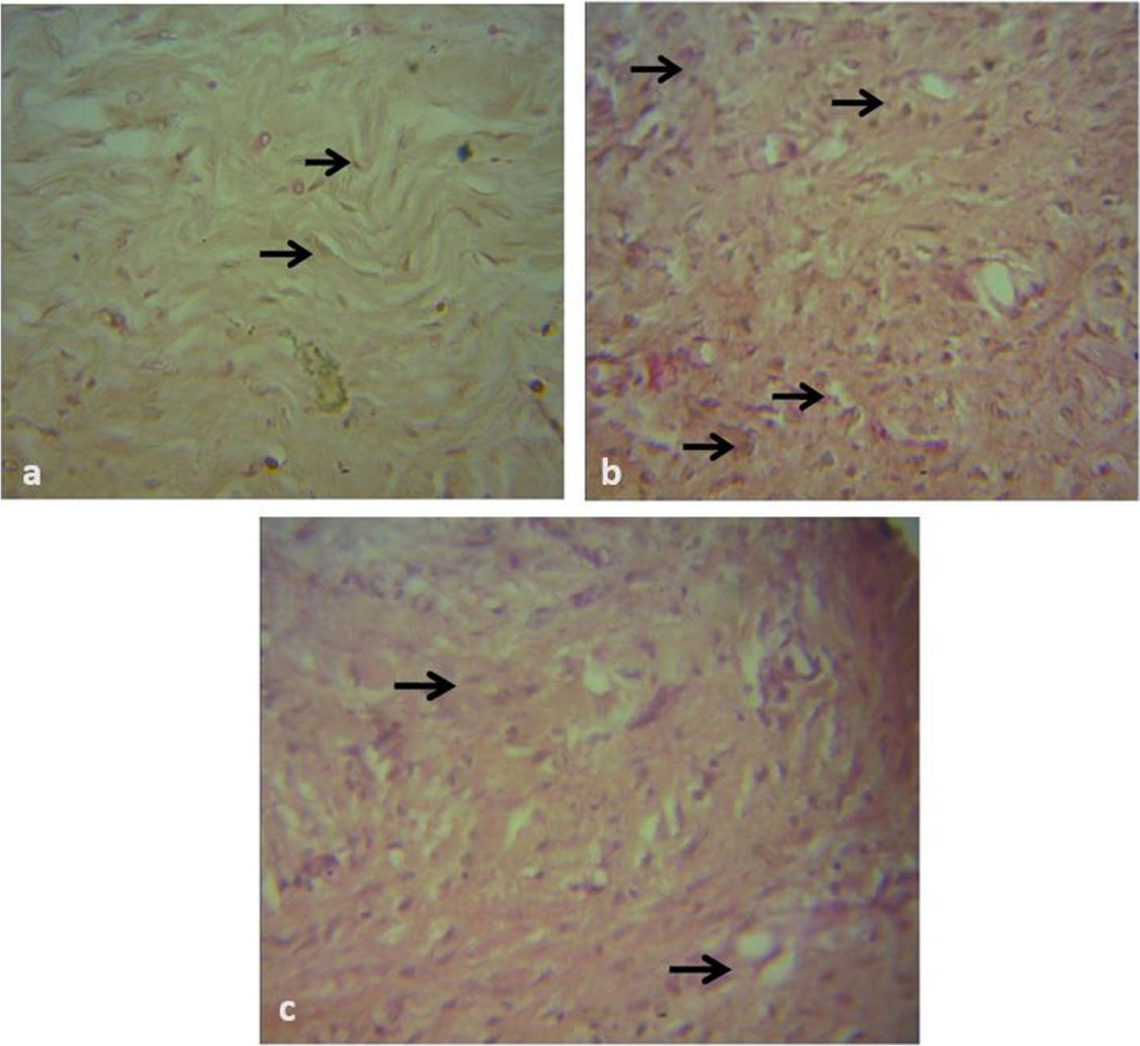
Photomicrograph of biopsy of meshed FTSG taken at day 10. H&E × 400. **a **There is sparse infiltration of fibrocytes seen (arrows) in the CON graft. **b ** There is mixed dense population of fibrocytes/fibroblasts (arrows) and collagen in the PRP graft. **c **There is dense population of fibrocytes (arrows) and collagen in the HON graft

**Fig. 8:**
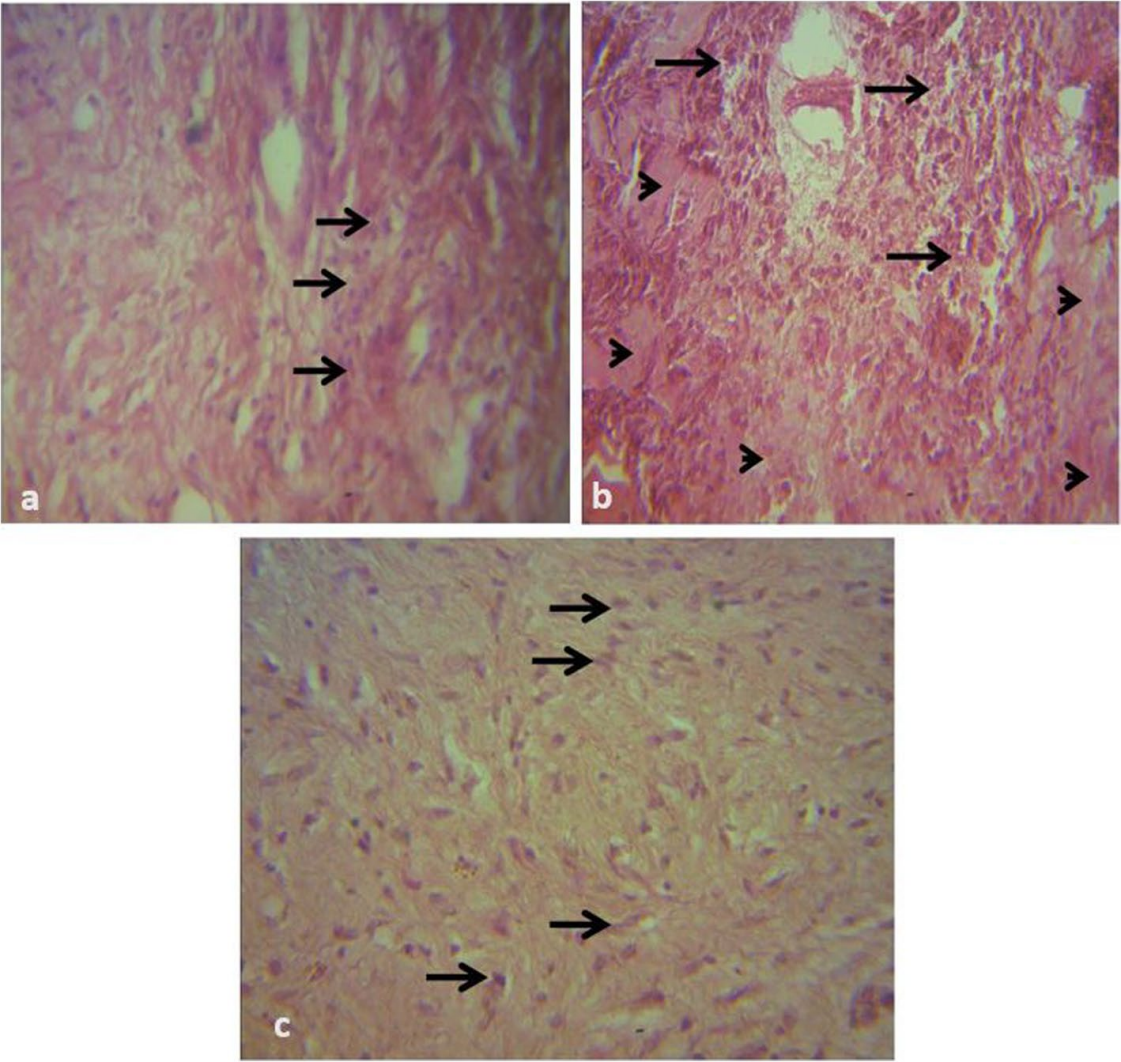
Photomicrograph of biopsy of meshed FTSG taken at day 14. H&E × 400. **a **There is a moderate cellular infiltration of the dermal connective tissue by macrophages (arrows) in the CON graft. **b **There is a diffuse moderate cellular infiltration of the dermis (arrows). The connective tissue is sparse, and the site appears oedematous (arrowheads) in the PRP graft. **c **There is mixed dense population of fibrocytes/fibroblasts (arrows) and collagen in the HON graft

**Fig. 9:**
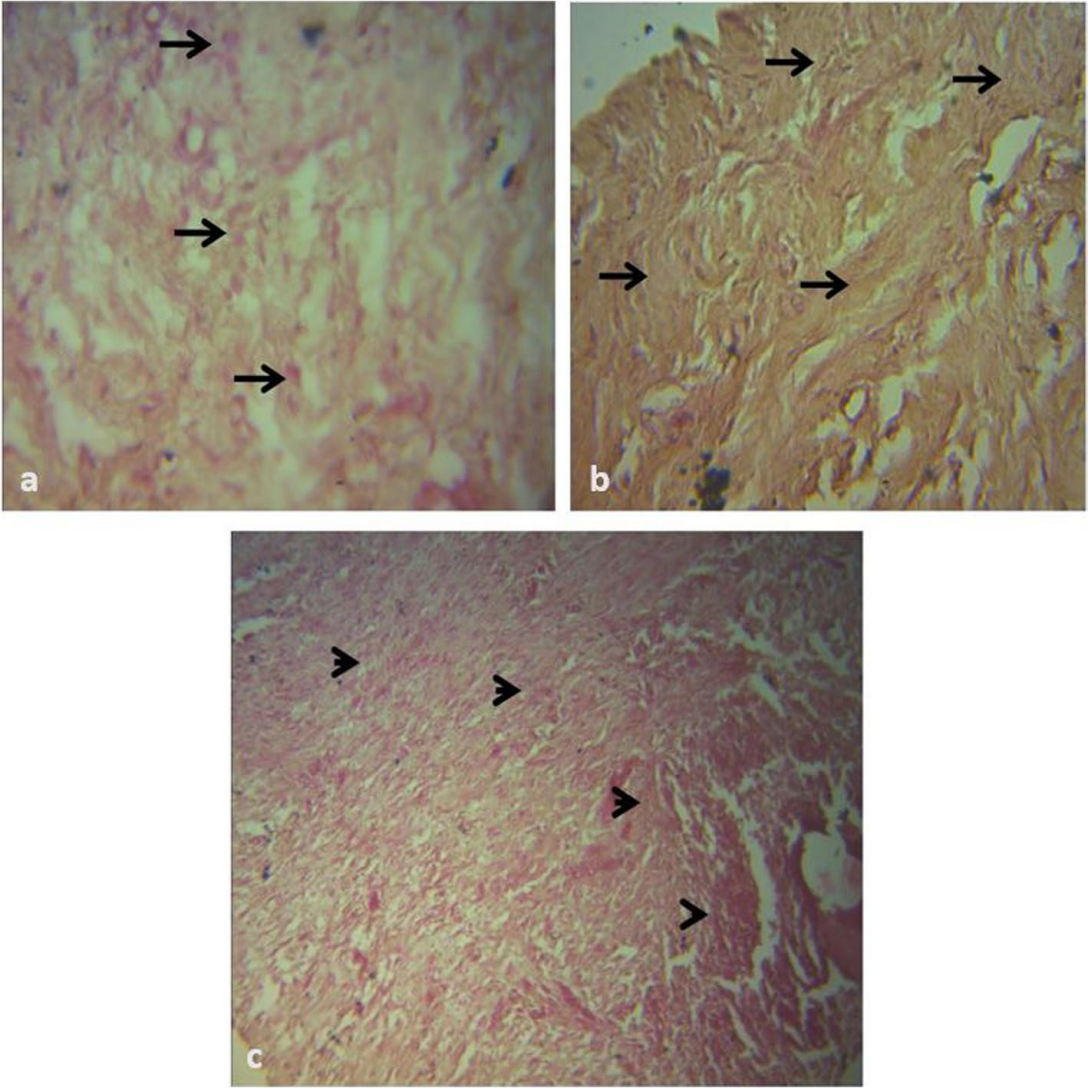
Photomicrograph of biopsy of meshed FTSG taken at day 21. H&E × 400. **a **The dermal connective tissue is sparse. There is a diffuse population of mostly fibroblasts (arrows) in the CON graft. **b **There is a dense collagen fibre laydown (arrows) in the PRP graft. **c **There is very dense collagen laydown (arrowheads) in the HON graft

Inflammatory cells, especially macrophages were observed from day 4 with a gradual decrease from day 4 to day 17. There was a gradual non-significant increase in fibroplasia and collagen laydown in all the groups from day 10 to 21 (Additional file). The HON and CON groups had marked macrophage infiltration without collagen laydown on day 4 while the PRP group had mild collagen laydown and moderate macrophage infiltration (Fig. 6). On day 10, there was mixed dense population of fibrocytes/fibroblasts and collagen in the PRP/HON groups and a sparse population of fibrocytes in the CON group (Fig. 7). A dense population of fibrocytes and collagen was observed on day 14 in the HON group with a moderate population of macrophages in the dermal connective tissue in the CON and PRP groups (Fig. 8). On day 21, the PRP and HON groups had better dermal connective tissue with the HON group having denser laydown of collagen fibres. The CON group, however, had sparse dermal connective tissue with a diffuse population of fibroblasts (Fig. 9).

### Graft Neovascularisation

Graft neovascularisation score was higher in the HON group in comparison to the CON and PRP groups at all biopsy time points except on Day 10 when both HON and PRP had a mean score of 1.0 (Additional file).

## Discussion

The results of this study show that honey enhanced graft take of meshed FTSGs by promoting early graft adherence, accelerating the rate neovascularisation and epithelisation, and facilitating good hair regrowth after healing.

In this study, graft take was classified as complete if there was full-thickness graft viability over more than three-quarters of the original (day 0) graft area on day 14 post-grafting (at suture removal) and partial where full-thickness graft viability was less than 3-quarters of the day 0 graft area and/or if there was epidermal sloughing [35]. Previous studies have adduced graft failure to infection, inadequate adherence and immobilisation of the grafted area, presence of seroma or haematoma between the graft and recipient bed, and slow revascularisation of the graft [6, 35, 36]. The observance of strict aseptic protocols during the grafting procedure and bandage change aided the prevention of graft wound infection in this study. This was also a core reason for the inclusion of 5-day post-grafting antibiotic therapy for all the groups [19, 20, 37, 38].

Graft adherence to the recipient bed is of utmost importance during the initial phases of graft healing before complete reattachment and revascularisation occurs. In this study, a higher percentage (83.3%) of non-mobile (adhered) grafts was observed in the HON group as compared with the PRP (57.1%) and CON (42.9%) groups at day 4. The first 48 to 72 h are very crucial to the survival of skin grafts, and the extent of adequate nutrition of the graft is solely dependent on how firmly adhered the graft is to the recipient bed [39, 40]. During this period, adherence to the recipient bed is necessary for the ‘kissing capillaries’ phenomenon where the open ends of deep dermal vessels touch those of the recipient bed and connect into one another (inosculation) [41]. The HON grafts’ better adherence may have been due to bee honey tissue adhesive potentials that have been well reported in previous studies [27, 28]. Bee honey is known to potentiate macrophage and fibroblast mobilisation into wounds [21, 42], which influenced the initial fibrin layer formed between the recipient bed and the grafted skin that aided the early graft adherence observed in the HON group. The volume of honey applied to the graft recipient bed is also very important. In this study, 1 drop (0.05 ml) per 3.0 cm × 1.5 cm wound area was effective in enhancing FTSG take and was applied between the graft meshes unto the recipient bed. This volume was decided following rigorous pilot study where larger volumes of honey were averse to graft take (unpublished data).

The presence of seroma or haematoma between the graft and recipient bed affects graft adherence and results in graft failure. The seroma creates a gap between the graft tissue and the recipient bed structures thus preventing plasmatic imbibition, capillary inosculation and revascularisation [20, 43]. In this study, seroma was noticed on day 4 for 2 each of the grafts in the PRP and HON groups which was absent by the next dressing change (day 7). On the other hand, the CON group had 3 grafts with seroma on day 4 with 1 persisting on day 7 and was later lost by day 10. The less seroma formation in the HON group may be associated with the graft tissue adherence potential of the Nigeria bee honey. In conventional grafting, bandaging aids minimally in seroma prevention. Currently, vacuum assisted closure (VAC) is advocated for seroma prevention following grafting [20, 43, 44].

Postoperative immobilisation is important to the graft healing process as it helps in reducing possible micro and macro movements that may disrupt the process of inosculation [7, 41]. Incorporating casts and splints into dressing skin grafts of the limbs, especially around joints [37, 41, 45, 46], the use of negative pressure bandaging [20, 43, 47, 48] and complete cage rest have been suggested in literature to aid immobilisation. Patient hospitalisation post-skin grafting for the first few days, or until suture removal have also been advocated [6, 7, 43]. The need to sedate or anaesthetise hyperactive patients in the first one week post grafting has also been suggested [41]. Immobilisation choices are often made through a combined assessment of patient status, size and location of the skin grafted sites, and financial concerns of the owners [41]. In this study, however, the dogs were kept on complete cage rest in smaller cages to prevent excessive movement and Elizabethan collars were placed to prevent access to the grafted area. In addition, all dressing changes till suture removal (day 14) were performed under sedation with 1 mg/kg of 2% Xylazine HCL administered IM [49]. It was observed that hyperactive dogs tended to have more partial graft take and graft failure than the relatively more docile and calm dogs. The temperament of the patient, as well as client compliance should therefore be taken into consideration when opting to use skin grafting for large wounds or defects and planning post-operative patient management.

Revascularisation after skin grafting is reported to occur as early as 22 h post-grafting and depends on adequate undisrupted adherence, a healthy and well-vascularised recipient bed and rate of neoangiogenesis [6, 39, 46, 50]. There is currently no consensus on whether FTSGs take better on fresh or granulating wounds [37], since both scenarios have been reported with good outcome [51]. However, most grafting procedures performed on fresh wounds were surgically created following tumour resections [37, 47, 52]. Large defects resulting from trauma are usually grafted after adequate decontamination, debridement and healthy granulation tissue formation [35]. A healthy granulating wound was selected as the ideal recipient bed for this study in an attempt to simulate the most common reason for which FTSGs will be indicated in our clime (i.e., trauma).

The rate of neovascularisation was assessed both qualitatively and quantitatively using graft colour and percent necrosis at each dressing change, respectively. Although there was no significant difference between groups for graft colour at all time points, clinically observable differences were notable at all dressing changes with the HON group returning more rapidly to a healthy pink colour (Table 1), indicative of faster neovascularisation in comparison to the PRP and CON groups. However, there was statistically significant decrease (*p* = 0.022) in percentage necrosis between the HON/PRP and CON groups on day 10, 14 and 17 (Fig. 2). The rapid neovascularisation of necrotic areas in the HON group, as well as the higher histological neovascularisation score in comparison to the CON and PRP groups on days 4, 14 and 21 is further evidence of the neoangiogenic properties of honey which have been well established in literature [21, 53, 54]. Some researchers have attributed this property of honey to be mediated via VEGF expression in the presence of vitamin C and hydrogen peroxide (H_2_O_2_) present in honey [25].

The rate of re-epithelisation of meshed grafts is an indication of the rate of healing [20] There was statistically significant decrease (*p* < 0.001) between the CON and HON groups on day 4, 7 and 10, and between the PRP/CON and HON groups on day 4 (*p* = 0.001) for percentage open mesh area. The increase in area of open mesh observed in the PRP and CON groups but not in the HON group on day 4 is worthy of note. Stanley et al. [20] observed a similar increase in open mesh area on day 7 in their control group (normal saline-treated grafts with conventional standard bolster dressing) when investigating the effect of negative pressure (vacuum) bandaging on FTSG take. It can therefore be inferred that the immediate decrease in open mesh area in the HON group may be as a result of early adherence and nutrition of the graft which prevented shrinkage of the skin tissue. Another study conducted to investigate the wound healing properties of UMF20 Manuka honey showed that the honey limited retraction of wound edges [55]. Nigeria bee honey appears to have a similar effect since there was immediate contraction of the open mesh edges of the grafts in contrast to retraction and widening of the open mesh spaces observed in the CON and PRP groups. The subsequent steady decrease in open mesh area could also be attributed to the epithelisation properties of honey [21, 33]. Healing in the CON group was observed grossly to be primarily as a result of re-epithelisation, whereas that of the PRP and HON groups were a combination of re-epithelisation and contraction. As a result, the HON grafts, especially had less interspersed epithelial lining which is better for hair regrowth and may have accounted for the better haircoat coverage as compared to the CON grafts.

Histologically, there was no significant difference between the treatment groups for HAIS and HRS (Additional file). However, it was observed that the PRP and HON groups had more fibroblast/fibrocytes proliferation in comparison to the CON group on day 10 (Fig. 7). By day 21, there was denser collagen laydown in the HON group in comparison with the PRP and CON groups (Fig. 9). This is worthy of note since one of the advantages of FTSGs to the veterinary surgeon is better trauma resistance. The collagen density of the tissue is directly proportional to its tensile strength [56–58], inferring that the grafts in the HON group will have better trauma resistance in comparison to those in the PRP and CON groups.

All grafts with complete or partial take were healed by day 21, assuming the original pigmentation of the donor skin (Fig. 1). Hence for good aesthetics, a donor site with skin pigmentation and hair coat colour matching that of the area around the recipient site is preferable. Also, attention should be paid when transplanting the donor skin, to conform the direction of hair to that of the area around the recipient site as much as possible [7]. 

Although, PRP had no graft loss in comparison to 1/6 graft loss for the HON group, analysis of the gross indices for the grafts with graft viability across all groups indicated that the grafts in the HON groups performed better than those in the PRP and CON groups for percentage adherence on day 4 and graft colour on all dressing days (except day 17). Grafts in the HON groups also had good hair regrowth, comparable to the PRP group. The extra cost involved with using PRP (vacuum tubes, centrifugation, etc.) and lack of constant electricity in most developing countries makes honey a better alternative for enhancing graft take.

## Conclusion

Nigeria bee honey enhances take of meshed full-thickness skin autografts, primarily within the first week of grafting, by promoting adherence of the graft to the recipient bed, neovascularisation, fibroblast proliferation and collagen laydown. Being readily available, honey can therefore be used as an alternative to more sophisticated and relatively more expensive modalities to enhance FTSG take.

## Methods

### Ethical approval

Ethical clearance (UI-ACUREC/19/0152) was obtained from the Animal Care and Use Research Ethics Committee (ACUREC) of the University of Ibadan, Nigeria. All methods were performed in accordance with the relevant guidelines and regulations stipulated in the ARRIVE guidelines.

### Experimental animals

Six apparently healthy entire adult male Nigerian indigenous dogs weighing 8 ± 2 kg with body condition scores 4–5 out of 9 were purchased from previous owners and housed in well-aerated dog kennels for the whole duration of the study. The dogs were acclimatised for 2 weeks before the commencement of the study, during which they were properly dewormed, vaccinated for rabies, treated for ectoparasites and conditioned to their housing, feeding and social enrichment routines. The dogs were fed with a home-cooked diet (consisting of rice, palm oil and fish, meat or eggs) once daily, and water was provided ad libitum. Social enrichment consisted of petting and communal interactions with one another once daily. The dogs were adjudged to be healthy based on clinical evaluation (physical examination, haematology and serum chemistry tests) before the commencement of the study.

### Study design

A simple randomised controlled experimental design was adopted for this study. Three of the 4 limbs (the 2 forelimbs and right hindlimb) of each dog were used. A total of 18 wounds were created, 1 wound per limb, and divided into 3 treatment groups (*n* = 6) (Table 2). Following wound creation, the limbs of each dog were coded and randomly assigned to one of the 3 treatments – Nigerian bee honey (test substance), platelet-rich plasma (positive control) or normal saline (negative control).

**Table 2:** Table showing experimental groups and description of treatments applied to the meshed FTSGs

Group	Treatment	Description
CON (*n* = 6)	0.2 ml normal saline	Negative Control
PRP (*n* = 6)	0.2 ml platelet-rich plasma	Positive Control
HON (*n* = 6)	0.05 ml honey	Test substance

Preparation Of platelet-rich plasma (PRP).

Platelet-rich plasma (PRP) was prepared according to a modified double centrifugation protocol described by Perazzi et al. [59]. Eight millilitres of venous blood was collected from the right lateral saphenous veins of each dog into sodium citrate vacuum tubes and centrifuged at 3000 rpm for 10 min. This separated the blood into plasma, buffy coat and red blood cell layers. The plasma was obtained from the tubes (approximately 4 ml) using a pipette and transferred into a sterile falcon tube, which was again centrifuged at 3000 rpm for 10 min. The result was an aggregation of the platelets at the bottom of the falcon tube and a platelet-poor plasma (PPP) supernatant. The PPP was discarded, leaving about 0.2 ml of plasma to reconstitute the platelets and form autologous PRP.

### Honey

Crude Nigeria bee honey was purchased from a local apiary in Ibadan, Oyo Sate, Nigeria. The honey was sieved with a fine muslin cloth to remove all bee remnants and stored in a tightly sealed container for use.

### Wound creation

#### Anaesthesia and Patient Preparation

Each dog was premedicated with 5% Tramadol (Tramacet, Ciron Drugs & Pharmaceuticals Pvt. Ltd., India), 0.1% Atropine Sulphate (Shanxi Shuguang Pharmaceutical Co. Ltd, China) and 2% Xylazine HCl (Xylased®, Bioveta, Czech Republic) at dosages of 5 mg/kg, 0.04 mg/kg and 2 mg/kg respectively via intramuscular (IM) injection. An intravenous (IV) line of Lactated Ringer’s solution (Hartmann’s Solution, Ashmina Ltd, Nigeria) was set at 5 ml/kg/hr via the left lateral saphenous vein using a 20G catheter. Anaesthesia was achieved and maintained with 5% Ketamine (Ketanir™, Aculife Healthcare Pvt. Ltd., India) at a dose of 10 mg/kg IM injection. The hair on two forelimbs and right hindlimb were clipped, aseptically prepared (by first cleaning thoroughly with soap and water then sterilising with alcohol and povidone iodine) and draped for surgery, from just above the elbows/hock to the proximal phalanges.

#### Surgical Procedure

With the dogs on left lateral recumbency, 3 cm × 1.5 cm full-thickness skin wounds were created on the lateral aspects of the right fore- and hindlimbs with a size 21 scalpel blade, using a sterile template, and the underlying subcutaneous fascia were removed to simulate a degloving injury. Following adequate wound irrigation, the wounds were dressed by an assistant and the dogs were turned over unto right lateral recumbency to repeat the procedure on the left forelimb.

#### Wound Care Before Grafting

The wounds were bandaged following creation. Bandaging consisted of a primary layer of sterile 5 cm x 3 cm gauze, a secondary layer of cotton padding and a tertiary layer of Crepe bandage held in place with circumferential strips of adhesive tape. Bandages were changed 24 h later and thereafter on alternate days for 5 to 6 days until adequate granulation tissues were formed after which the wounds were grafted.

### Grafting

#### Recipient Bed Preparation

Following anaesthesia and patient positioning on lateral recumbency, the wound beds were prepared by creating fresh bleeding edges and undermining the skin around the wound through blunt-sharp dissection with Mayo scissors prior to harvesting of skin from the donor site. The wound was thoroughly irrigated with normal saline and covered with a saline-moistened sterile gauze while the donor skin was prepared and harvested.

#### Harvesting of Donor Skin

**Fig. 10:**
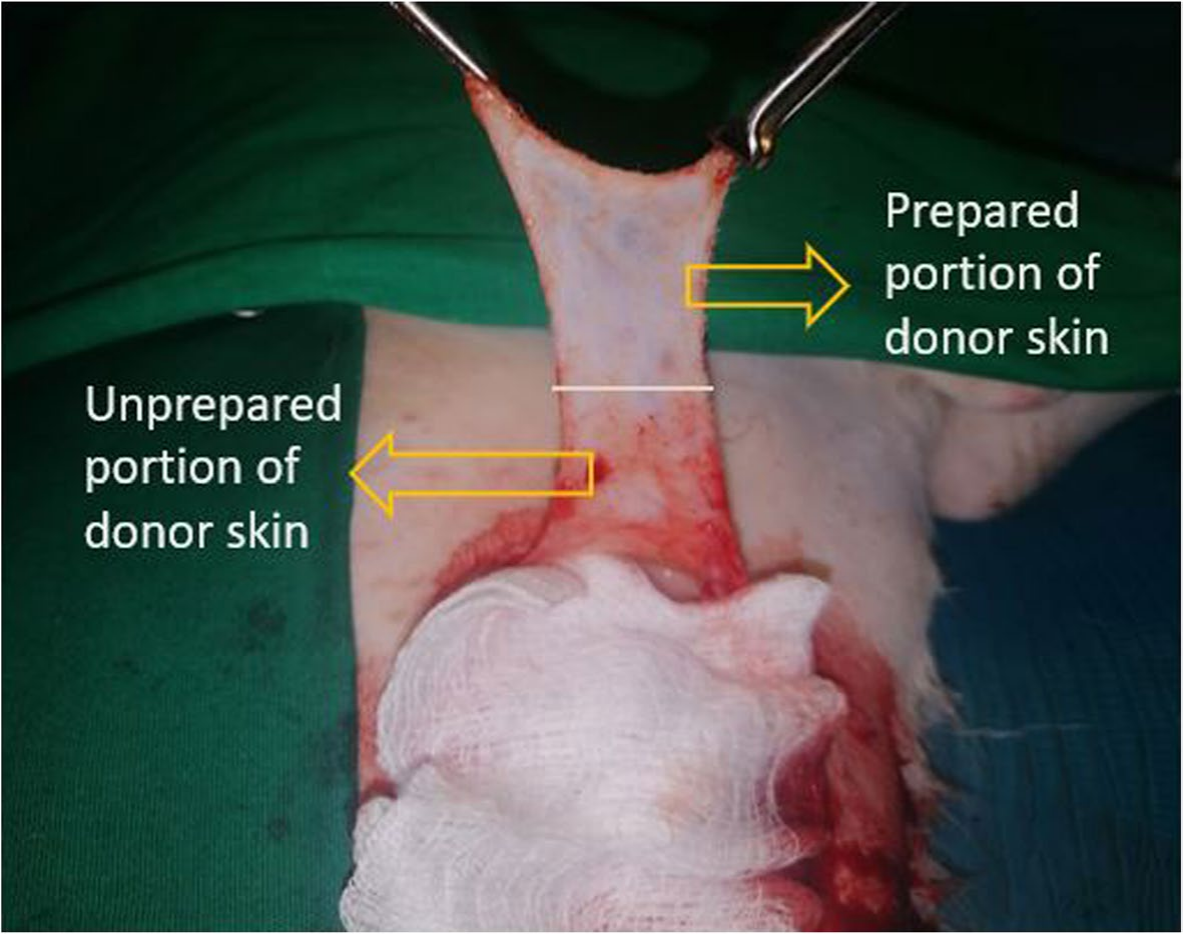
Image of the donor skin being harvested from the lateral thorax. The difference between the prepared (cobblestone appearance with visible hair follicles) and unprepared portions of the donor skin is clearly demarcated

Donor skin harvesting consisted of using a sterile template to mark a 9 cm × 4.5 cm area of skin on the right lateral thorax while paying attention to hair direction and colour. Three sides of the demarcated area were incised using a scalpel blade. The resultant flap of skin was elevated by grasping the two vertices with Allis tissue forceps and separating the skin from the subcutaneous fascia via blunt-sharp dissection using scissors. The elevated skin flap was prepared for free skin grafting by meticulously removing all subcutaneous fascia and attached sub‐dermal remnants with scissors until the hair follicles were visible (cobblestone appearance) (Fig. 10).

#### Grafting Procedure

**Fig. 11:**
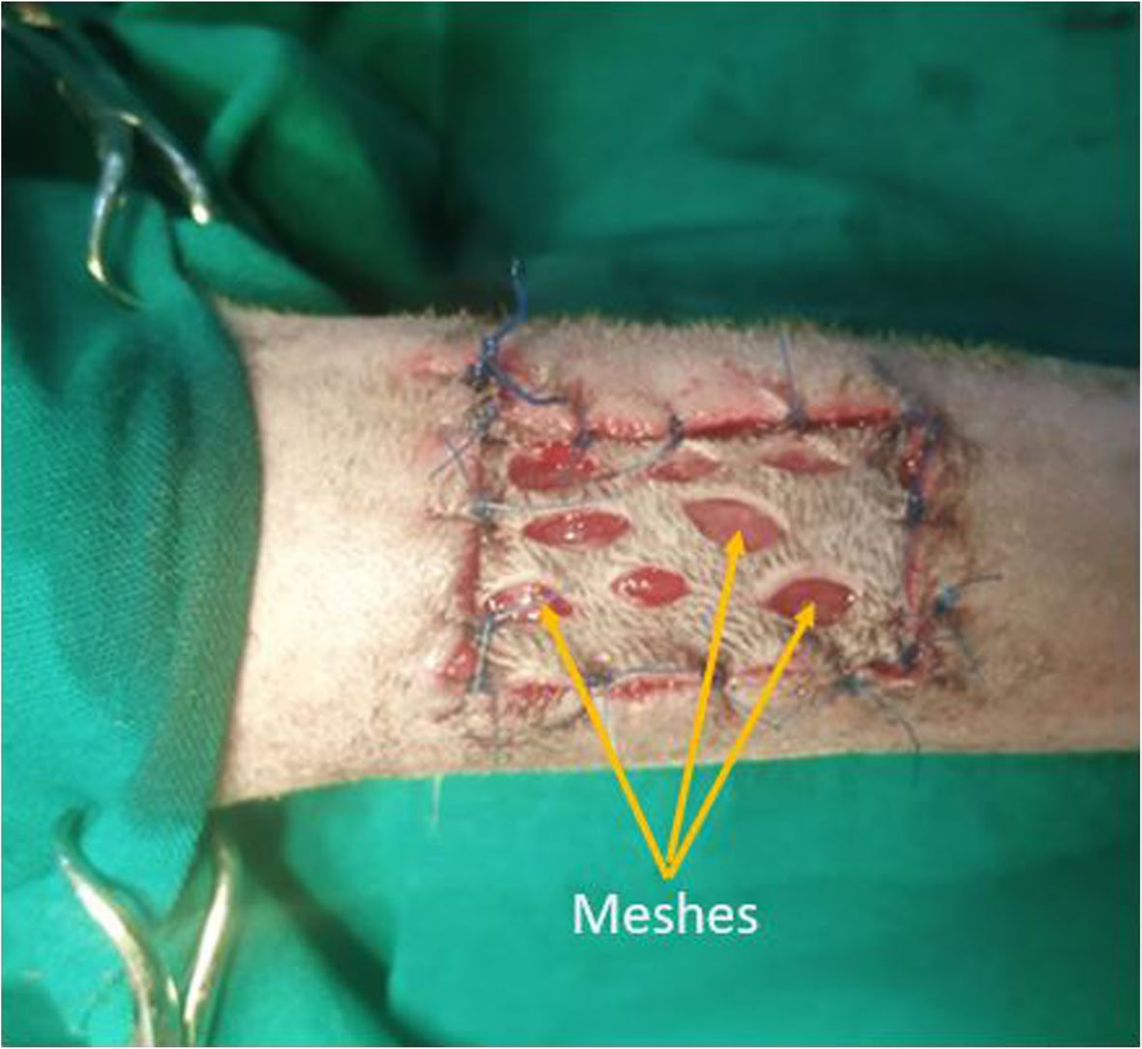
Image of a graft secured to the recipient bed. Meshed full thickness cutaneous autografts were secured to the wound edges of the recipient sites with simple interrupted sutures using size 3/0 nylon

The grafting procedure for each limb commenced with excising a 3 cm × 1.5 cm skin section from the elevated flap, and making 8 parallel stab incisions using a size 11 scalpel blade (meshing) as previously described [20]. Each meshed skin section was transferred to the prepared recipient bed and secured to the wound edges with 14 simple interrupted sutures using size 3/0 nylon (Huaiyin Medical Instruments Co. Ltd, China) (Fig. 11). Each wound was irrigated, the assigned treatment applied, and the wound dressed. The defect on the donor site was repaired with size 1 nylon (Huaiyin Medical Instruments Co. Ltd, China) in a horizontal mattress suture pattern.

### Treatment application and dressing

Following sterilisation of the skin around each wound with povidone-iodine solution, treatments were applied to the assigned wounds by dropping the required volume (0.05 ml of honey, 0.2 ml of PRP or 0.2 ml of normal saline) at the open mesh area with a 1 ml syringe and spreading it beneath the graft to form a thin layer between the donor skin and the recipient bed on day 0 of grafting prior to wound dressing. Wound dressing consisted of a single primary layer of non-adherent petrolatum-impregnated tulle gras and normal gauze, a secondary layer of cotton padding, and a tertiary layer of Crepe bandage taped with Pet Flex no chew veterinary adhesive bandage (Andover Healthcare, USA). The first dressing change was at day 4 after skin grafting and repeated on days 7, 10, 14, 17 and 21. Treatments were applied (day 4, 7, 14 and 17) on the surface of grafted area before wound dressing. Sutures for both the grafted wound and donor sites were removed on day 14 post-grafting.

### Antibiotic and analgesic therapy

Amoxicillin (CP-Cillin, Swiss Parenterals Ltd., India) was administered at 15 mg/kg IM injection twice daily for 5 days. Pain was controlled with 5% Tramadol at a dosage of 5 mg/kg via IM injection once daily for 3 days.

### Evaluation of graft take

Qualitative (graft adherence, graft health evaluation, graft colour and hair regrowth) and quantitative (rate of wound epithelisation and neovascularisation) assessment protocols, including histopathological evaluation were used. Graft take was defined as complete (where there was full-thickness graft viability over more than three-quarters of the day 0 graft area on day 14 post-grafting), partial (where full-thickness graft viability was less than 3-quarters of the day 0 graft area and/or if there was epidermal sloughing) and lost (where there was complete loss of the graft tissue).

#### Assessment of Graft Adherence

Graft adherence was assessed on Day 4 post-grafting as previously described [20] by applying gentle digital pressure on the graft and pushing laterally, attempting to slide the graft over the bed.

#### Graft Health Evaluation

A modified graft health evaluation form was used to record wound exudation, colour and nature, appearance of secondary dressing layer, graft oedema and hydration, and appearance of the skin around the wound at each dressing change. Seromas that developed under the grafts were treated by aspirating the fluid using a 30-gauge 1 ml syringe. Digital photographs were also taken at time of dressing, and qualitative assessments (scoring) of graft colour (Table 3) were made from a review of the images by an investigator blinded to the randomisation.

**Table 3:** Five-point scale used for assessment of graft colour of meshed FTSGs at dressing changes

Score	Description
1	Healthy pink
2	Mottled pink
3	Mottled, purple
4	Dark purple or black
5	Slimy white

#### Assessment of Rate of Neovascularisation and Epithelisation

Wound measurements were performed with downloadable software (Digimizer Version 5.6.0, MedCalc Software Ltd., http://www.digimizer.com) using the digital photographs taken at each dressing. The total area of graft (cm^2^), total area of graft necrosis (cm^2^) and total area of open mesh (cm^2^) were measured and used to determine the percentage necrosis and open mesh area using Eqs. 1 and 2 respectively. Necrosis in this study was defined as dark/black discoloration of the grafted donor skin [20].

1 $$\mathrm{Percent}\;\mathrm{necrosis}\;=\frac{Area\;of\;necrosis\;(Day\;n)}{Total\;area\;of\;graft\;(Day\;n)}\times100.$$

2 $$\mathrm{Percent}\;\mathrm{open}\;\mathrm{mesh}\;\mathrm{area}\;=\;\frac{Area\mathit\;of\mathit\;open\mathit\;mesh\mathit\;\mathit(Day\mathit\;n\mathit)}{Area\mathit\;of\mathit\;open\mathit\;mesh\mathit\;\mathit(Day\mathit\;0\mathit)}\times100.$$

#### Grafted Skin Biopsy Collection and Histologic Evaluation

Biopsies were obtained from the corner of each grafted wound with a 3 mm disposable dermal biopsy punch in a systematic clockwise pattern on day 4, 10, 14, and 21. Obtained samples were fixed immediately in 10% neutral buffered formalin. Representative sections were stained with haematoxylin and eosin (H&E) for microscopic evaluation of inflammation and repair using a modified semi-quantitative scoring system [60, 61].

To evaluate inflammation, inflammatory cell infiltration and degree of haemorrhage and necrosis were scored as follows: 0 = none, 1 = minimal, 2 = moderate, and 3 = marked. The individual scores were then summed to formulate a histologic acute inflammation score (HAIS) with range 0–9. For tissue repair, fibroblast proliferation, collagen density and neovascularisation were compared with the day 0 sample and scored as follows: 0 = absent, 1 = mild, 2 = moderate and 3 = marked. These were also summed to formulate a histologic repair score (HRS) with range 0–9.

### Data analysis

Numerical variables (percentage open mesh area and percentage necrosis) were expressed as mean ± standard error while the non-numerical variables were expressed as ordinal scores. Statistical significance between groups was assessed using 3 factor ANOVA with the fixed factors of group and time and the random factor of dog with SPSS v16 for windows. Statistically significant differences (*p* < 0.05) were followed by post-hoc multiple comparison test.

## Supplementary Information

**Additional file 1. **Mean results of semi-quantitative Histologic Acute Inflammation Score (HAIS) and Histologic Repair Score (HRS).

## Data Availability

The datasets used and/or analysed during the current study are available from the corresponding author on reasonable request.

## Abbreviations

FTSG: Full thickness skin graft
H&E: Haematoxylin & eosin
HAIS: Histologic acute inflammation score
HRS: Histologic repair score
PRP: Platelet-rich plasma
STSG: Split thickness skin graft
