# Supplementary material for: Nigeria bee honey-enhanced adherence, neovascularisation and epithelisation of full-thickness skin autografts on distal extremities of dogs
Source: BMC Vet Res. 2022 Mar 11;18:94. doi: 10.1186/s12917-022-03192-w (PMC8915464; doi:10.1186/s12917-022-03192-w)
Supplement: Supplementary file 1 — Additional file 1. Mean results of semi-quantitative Histologic Acute Inflammation Score (HAIS) and Histologic Repair Score (HRS). [file 12917_2022_3192_MOESM1_ESM.docx]

Additional file

Mean results of semi-quantitative Histologic Acute Inflammation Score (HAIS) and Histologic Repair Score (HRS).

| Treatment  Group | Histologic Parameters | | | | | | | |
| --- | --- | --- | --- | --- | --- | --- | --- | --- |
|  | Inflammatory cell | Haemorrhage | Necrosis | HAIS | Fibroblast proliferation | Collagen density | Neovascularisation | HRS |
| DAY 4 | | | | | | | | |
| CON | 2.3 | 1.3 | 2.8 | 6.3 | 0.9 | 0.8 | 0.0 | 1.7 |
| PRP | 1.8 | 1.0 | 2.6 | 5.4 | 1.2 | 1.0 | 0.2 | 2.4 |
| HON | 2.0 | 1.0 | 2.7 | 5.7 | 0.7 | 1.0 | **0.3** | 2.0 |
| DAY 10 | | | | | | | | |
| CON | 2.0 | 0.6 | 1.4 | 4.0 | 1.8 | 2.0 | 0.8 | 4.6 |
| PRP | 2.2 | 0.4 | 1.4 | 4.0 | 2.8 | 2.0 | **1.0** | 5.8 |
| HON | 1.8 | 0.3 | 1.5 | 3.5 | 2.3 | 2.0 | **1.0** | 5.3 |
| DAY 14 | | | | | | | | |
| CON | 2.0 | 0.8 | 1.5 | 4.3 | 2.5 | 1.5 | 1.1 | 5.1 |
| PRP | 2.0 | 1.0 | 1.7 | 4.7 | 2.3 | 1.7 | 1.3 | 5.3 |
| HON | 2.2 | 1.0 | 0.8 | 4.0 | 2.2 | **2.2** | **1.4** | 5.8 |
| DAY 21 | | | | | | | | |
| CON | 1.2 | 0.2 | 0.4 | 1.8 | 1.6 | 2.6 | 1.6 | 5.8 |
| PRP | 1.0 | 0.0 | 0.7 | 1.7 | 1.5 | 2.8 | 1.8 | 6.0 |
| HON | 1.0 | 0.3 | 0.0 | 1.3 | 1.3 | **3.0** | **2.0** | 6.3 |
